# Association between depression and HIV infection vulnerable populations in United States adults: a cross-sectional analysis of NHANES from 1999 to 2018

**DOI:** 10.3389/fpubh.2023.1146318

**Published:** 2023-06-01

**Authors:** Yan Xu, Yuexin Huang, Jie Peng, Ruiti Tang, Bin Luo, Zhiwei Xia

**Affiliations:** ^1^Department of Clinical Laboratory, Hunan Aerospace Hospital, Changsha, Hunan, China; ^2^Department of Urology, Hunan Aerospace Hospital, Changsha, Hunan, China; ^3^Department of Neurology, Aerospace Center Hospital, Beijing, China; ^4^Department of Neurology, Hunan Aerospace Hospital, Changsha, Hunan, China

**Keywords:** HIV, depression, HIV infection vulnerable populations, HIV disclosure, NHANES

## Abstract

**Background:**

Although the government has made a commitment to advance education on HIV disclosure, depression continues to play a significant role in whether people living with HIV (PLWH) choose to disclose their HIV status to families or friends. Vulnerable populations who are at risk of contracting HIV may also be more susceptible to mental illness. However, there is a limited understanding of the association between depression and vulnerable populations affects by HIV among United States adults. We aimed to explore the incidence of depression in the HIV infection vulnerable populations and assessed the association between the HIV infection vulnerable populations and depression.

**Methods:**

We analyzed the most current statistics from the National Health and Nutrition Examination Survey (NHANES) that included 16,584 participants aged 18 years or older between 1999 and 2018. The Patient Health Questionnaire-9 (PHQ-9) was used to evaluate symptoms of depressive disorder. Demographic characteristics were compared between the HIV infection vulnerable groups and HIV infection low-risk groups. Multivariable logistic regression analysis was also carried out to evaluate the odds rate and association between the HIV infection vulnerable populations and depression.

**Results:**

Based on the most recent statistics from NHANES, HIV infection vulnerable populations were male, younger, less married or living together, non-Hispanic White people, lower income, and lower body mass index (BMI), with higher levels of cigarette smoking, alcohol drinking, a higher prevalence of depression, lower prevalence of hypertension and diabetes mellitus (DM; *p* < 0.05). Additionally, individuals with severe depression had a higher prevalence of cardiovascular disease (CVD), hypertension, DM, chronic kidney disease (CKD), and a higher proportion of HIV infection vulnerable populations and less married or living together (*p* < 0.01). Finally, the odds of depression from the logistic regression were significantly increased in HIV infection vulnerable groups (*p* < 0.01).

**Conclusion:**

Depression might be associated with HIV infection vulnerable populations in the United States adults. More research is needed to evaluate the association between HIV infection vulnerable populations and depression and explore their causal associations. In addition, prevention efforts focusing on HIV disclosure and HIV infection vulnerable populations in the United States should address common co-prevalent depression to reduce new HIV infections.

## Introduction

Vulnerable populations with human immunodeficiency virus (HIV) infections have experienced more high-risk HIV behaviors, which cause morbidity, mortality, and disability in the United State (1–3). These groups reported higher rates of substance use, alcohol abuse, smoking, avoidant health behavior, condomless sex, being Black/African American, gay and sex workers, and with less income and other affective disorders than the normal populations (1, 4–6). They also have poor mental health and a low socioeconomic status (1, 7). Therefore, HIV infection vulnerable populations may suffer long-term health concerns in the future, especially when infected with HIV.

With the significant advancements in the preclusion and the modern combination of antiretroviral therapy (ART), HIV infection had become a chronic complaint with an ordinary life expectancy in developed countries. The prevalence of new HIV-infected individuals has decreased, and the number of people living with HIV (PLWH) has not markedly increased over the past decade (1, 8). Globally, more than 37.9 million experienced HIV infection, and in the United States, the number of PLWH increased by 33% from 0.80 million (2009) to 1.02 million (2018) (9, 10). Despite the indication that HIV infection carries a higher risk of developing depression, the mechanisms responsible for the increased risk have not been completely documented. Nonetheless, previous studies had focused only on HIV infection and the use of antiretroviral treatment substances for noninfectious diseases (9).

Depression, a kind of mental health problem represented by various symptoms, including low mood or energy, lack of interest or concentration, anxiety, guilt, and changes in appetite and psychomotor activity. Depression is a significant reason for suicide and disease burden, and is recognized as an obstacle to implementing antiretroviral therapy (ART) for PLWH, increasing the spread of HIV and sexually transmitted diseases (6, 11–14). It is still plays an important role in whether PLWH decides to disclose their HIV status to families or friends (15). The current epidemiological research shows that adverse mental health conditions increase the risk of HIV infections (16). In addition, previous studies have explored the social conditions and the diseases in order to investigate the association between HIV high-risk behaviors and depression in specific population such as men who had sex with men (MSM), transgender women, sex workers, etc., and found that increasing HIV high-risk behaviors was associated with depression (2, 6, 16–18). However, limited studies have estimated the association between depression and HIV infection in vulnerable populations, especially in the United States (19, 20). This cross-sectional survey aimed to review and evaluate the association between depression and the HIV infection vulnerable populations in the United States by adopting statistics from the NHANES from 1999 to 2018.

## Materials and methods

### Study population

**Figure 1:**
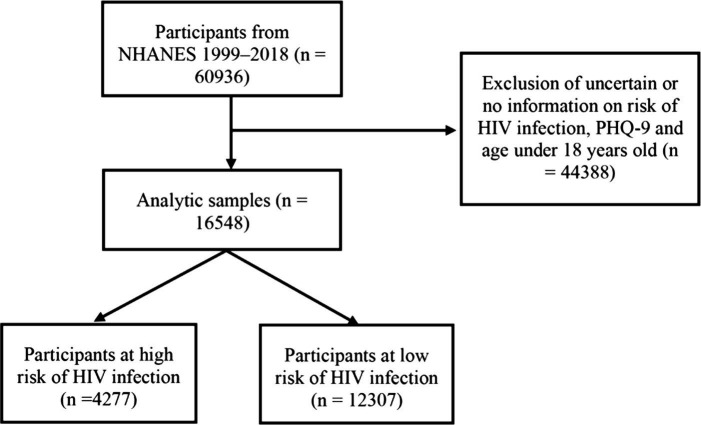
Flow chart of study participants. Sample selection and exclusion criteria for comparing high-risk of HIV infection and low-risk of HIV infection participants. NHANES, National Health and Nutrition Examination Survey; PHQ-9, the patient health questionnare-9.

The NHANES is managed by the Centers for Disease Control and Prevention to assess the health of the United States population adopting a representative population sample of United States civilian community residence members, adopting a complex, multistage, possibility-sampling plan. The research was conducted regularly before 1999 and continuously after that. Elements of data collection and the survey design have been formerly represented (21). Among 60,936 participants, the analysis excluded those with uncertain or no information on the risk of HIV infection, information on PHQ-9, and those under 18 years old. Finally, 16,584 participants from NHANES were included (Figure 1). This study was based on secondary data analysis that was shortened by personal identifiers and did not require institutional review. Interviews were conducted to gather information on factors that increase the risks related to HIV acquisition and transmissions such as unprotected sexual behaviors involving rectal or urethral contact, self-reported history of sexually transmitted diseases, injected substance use, and adverse mental health conditions. Therefore, only individuals aged 18 years or older who had not been infected with HIV were included in this research, representing United States adults.

### Data collection

In every 2 years survey, participants completed in-house interviews and went to a mobile testing center that they replied to extra questionnaires and underwent physical inspection and blood sample gathering. A standardized survey was adopted to collect data on age, gender, race/ethnicity, BMI, family income-to-poverty ratio (PRI), smoking status, alcohol drinking status, education level, and self-reported history of depression, hypertension, CVD, CKD, and DM (22). Age and BMI were defined as continuous variables. Categorical variables were as follows: race/ethnicity (Mexican American, non-Hispanic White, non-Hispanic Black, other Hispanic, or other), sex (male, female), PRI (<130%, 130–350%, or > 350%), marital status (never married, married or living together, and widowed/divorced/separated), education status (high school or less, more than high school), alcohol drinking status (former drinking, heavy drinking, moderate drinking, mild drinking, or never drinking), history of hypertension (no, yes), history of CVD (no, yes), history of CKD (no, yes), and history of DM (no, diabetes mellitus, impaired fasting glucose, and impaired glucose tolerance).

In addition, the PHQ-9 was applied to assess depressive status during the face-to-face interviews (23). These nine symptoms were (a) anhedonia, (b) depressed emotion, (c) sleep trouble, (d) fatigue, (e) changes in appetite, (f) low self-esteem, (g) concentration troubles, (h) psychomotor problems, and (i) suicidal thoughts. Each symptom item in PHQ-9 was scored on a 0–3 calibration, from 0 (“not any”) to 3 (“almost every day”). The entire score ranged from 0 to 27, with scores ≥10 symbolizing clinically meaningful depressive symptoms. Furthermore, this study classified participants into the following five groups based on their total scores: 0–4 (no depression), 5–9 (mild depression), 10–14 (moderate depression), 15–19 (moderately severe depression), and 20–27(severe depression) (24, 25). The PHQ-9 is proven to measure depressive symptom severity (26), and the total score was evaluated as a constant variable to further confirm the results.

The NHANES gathers information on the risk behaviors of HIV acquisition and transmission; the risk behaviors including condomless sex, anal intercourse, self-reported history of sexually transmitted diseases, injected substance use, and adverse mental health conditions. Participants were defined as HIV infection vulnerable groups base on if they had any one of the following high-risk behaviors: multiple sex partners in the last year, self-reported diagnoses of any sexually spread infections (such as gonorrhea, genital warts, genital herpes, and chlamydia), and drug addicts (7, 27, 28). The rest participants were classified as the HIV infection low-risk group.

### Statistical analysis

According to the NHANES statistical analysis guidelines, all analyses were applied to weighted samples and considered the design’s stratification and clustering to derive assessments that applied to United States adults. In descriptively statistical analysis, constant variables were demonstrated in the mean and SD, and categorical variables were depicted in the form of frequency and percentage. The *t*-test and chi-squared test compared mean values and categorical variables, respectively. In addition, multiple logistic regression models evaluated the associations between depression and the HIV infection vulnerable populations after adjusting demographic covariates and a history of chronic diseases. Model 1 adjusted for sex and age; Model 2 additionally adjusted for poverty income ratio status, race/ethnicity, BMI, smoking, marital status and drinking status; and model 3 further adjusted for history of CVD, hypertension, DM, and CKD. This research assessed adjusted odds ratios (ORs) for depression according to HIV risk status to evaluate the possible association between depression and HIV risk status. All statistical analyses were performed for sampling weights using R software (version 4.1), with value of *p* not more than 0.05.

## Results

### Population characteristics by depression categories

This study included 60,936 individuals from the NHANES database (1999–2018). After excluding participants without information on the risk of HIV infection, information on PHQ-9, and age under 18 years old, the analysis included 16,584 individuals, of which 1,609 (9.7%) had clinically significant depressive symptoms. They were then divided into two groups: (i) HIV infection low-risk groups (*n* = 12,307) and (ii) the HIV infection vulnerable groups (*n* = 4,277; Figure 1). Participants were classified into five groups to assess the correlation between depression and HIV infection vulnerable populations. These were no depression, mild depression, moderate depression, moderately severe depression, and severe depression groups (Table 1). Relative to lower PHQ-9 scores groups, individuals with moderately severe depression and severe depression groups were older, with 73.2% and 70.3% female. These two groups had predominantly non-Hispanic White people, with less income, less education, less married or living together, higher BMI, and higher smoking and alcohol drinking rates (*p* < 0.01). In addition, individuals in moderately severe depression and severe depression groups had a higher prevalence of CVD, hypertension, CKD and DM. They also occupied a higher proportion of the HIV infection vulnerable populations (*p* < 0.01).

**Table 1:** Characteristics of participants by depression categories in the National Health and Nutrition Examination Survey (NHANES), 1999–2018.

Characteristics	No depression	Mild depression	Moderate depression	Moderately severe depression	Severe depression	*p* value	*p* trend^#^
	*N = 12,221*	*N = 2,754*	*N = 988*	*N = 429*	*N = 192*		
Age, years, mean (SD)	37.8 (11.8)	37.8 (12.2)	38.9 (12.1)	40.9 (11.7)	42.9 (10.8)	<0.001	<0.001
Gender, *N* (%)						<0.001	0
Female	6,786 (55.5%)	1,775 (64.5%)	696 (70.4%)	314 (73.2%)	135 (70.3%)		
Male	5,435 (44.5%)	979 (35.5%)	292 (29.6%)	115 (26.8%)	57 (29.7%)		
HIV risk status, *N* (%)						<0.001	0
HIV high risk	2,842 (23.3%)	878 (31.9%)	336 (34.0%)	144 (33.6%)	77 (40.1%)		
HIV low risk	9,379 (76.7%)	1,876 (68.1%)	652 (66.0%)	285 (66.4%)	115 (59.9%)		
Family income-to-poverty ratio, *N* (%)						<0.001	0
<130%	3,299 (29.0%)	1,013 (39.4%)	481 (51.9%)	229 (56.7%)	102 (58.0%)		
>350%	4,047 (35.6%)	605 (23.6%)	143 (15.4%)	48 (11.9%)	20 (11.4%)		
130 to <350%	4,026 (35.4%)	951 (37.0%)	302 (32.6%)	127 (31.4%)	54 (30.7%)		
Race and ethnicity, *N* (%)						<0.001	<0.001
Non-Hispanic white	4,850 (39.7%)	1,124 (40.8%)	418 (42.3%)	192 (44.8%)	88 (45.8%)		
Non-Hispanic black	2,647 (21.7%)	614 (22.3%)	241 (24.4%)	92 (21.4%)	36 (18.8%)		
Mexican American	2,129 (17.4%)	459 (16.7%)	137 (13.9%)	63 (14.7%)	18 (9.38%)		
Other Hispanic	1,177 (9.63%)	286 (10.4%)	118 (11.9%)	57 (13.3%)	35 (18.2%)		
Other	1,418 (11.6%)	271 (9.84%)	74 (7.49%)	25 (5.83%)	15 (7.81%)		
Marital status, *N* (%)						<0.001	0
Married/living together	7,492 (64.5%)	1,409 (54.4%)	455 (48.1%)	179 (43.2%)	83 (43.9%)		
Never married	2,611 (22.5%)	688 (26.6%)	248 (26.2%)	105 (25.4%)	40 (21.2%)		
Widowed/divorced/separated	1,511 (13.0%)	492 (19.0%)	243 (25.7%)	130 (31.4%)	66 (34.9%)		
Education, *N* (%)						<0.001	0
High school or less	4,955 (40.5%)	1,346 (48.9%)	531 (53.7%)	253 (59.0%)	118 (61.5%)		
More than high school	7,266 (59.5%)	1,408 (51.1%)	457 (46.3%)	176 (41.0%)	74 (38.5%)		
BMI, kg/m^2^, mean (SD)	28.6 (6.85)	30.0 (8.10)	30.5 (8.69)	32.0 (9.34)	31.8 (8.32)	<0.001	<0.001
Cerebrovascular disease status, *N* (%)						<0.001	0
No	11,304 (97.3%)	2,430 (93.8%)	858 (90.6%)	352 (85.0%)	152 (80.4%)		
Yes	316 (2.72%)	160 (6.18%)	89 (9.40%)	62 (15.0%)	37 (19.6%)		
Hypertension status, *N* (%)						<0.001	0
No	9,316 (76.2%)	1,908 (69.3%)	626 (63.4%)	237 (55.2%)	95 (49.5%)		
Yes	2,905 (23.8%)	846 (30.7%)	362 (36.6%)	192 (44.8%)	97 (50.5%)		
Diabetes mellitus status, *N* (%)						<0.001	0
Diabetes mellitus	1,071 (9.06%)	329 (12.4%)	154 (16.1%)	72 (17.1%)	45 (23.8%)		
Impaired fasting glucose	385 (3.26%)	93 (3.51%)	24 (2.52%)	9 (2.14%)	6 (3.17%)		
Impaired glucose tolerance	403 (3.41%)	98 (3.70%)	31 (3.25%)	21 (4.99%)	9 (4.76%)		
No	9,959 (84.3%)	2,132 (80.4%)	745 (78.1%)	319 (75.8%)	129 (68.3%)		
Chronic kidney diseases status, *N* (%)						<0.001	<0.001
No	10,691 (91.8%)	2,348 (89.1%)	831 (87.4%)	343 (85.1%)	153 (83.6%)		
Yes	957 (8.22%)	287 (10.9%)	120 (12.6%)	60 (14.9%)	30 (16.4%)		
Smoking status, *N* (%)						<0.001	0
No	9,526 (80.0%)	1,826 (68.8%)	555 (57.4%)	222 (52.9%)	92 (48.2%)		
Yes	2,377 (20.0%)	830 (31.2%)	412 (42.6%)	198 (47.1%)	99 (51.8%)		
Alcohol drinking status, *N* (%)						<0.001	0
Former drinker	1,458 (12.1%)	381 (14.1%)	166 (17.0%)	82 (19.4%)	40 (20.8%)		
Heavy drinker	3,103 (25.7%)	843 (31.2%)	311 (31.8%)	129 (30.5%)	74 (38.5%)		
Moderate drinker	2,174 (18.0%)	505 (18.7%)	173 (17.7%)	77 (18.2%)	30 (15.6%)		
Mild drinker	3,729 (30.9%)	692 (25.6%)	223 (22.8%)	94 (22.2%)	31 (16.1%)		
Never drinker	1,608 (13.3%)	277 (10.3%)	104 (10.6%)	41 (9.69%)	17 (8.85%)		

Data were presented as unweighted frequencies and proportions.

# Test for trend based on the variables containing median values for each quintile.

### Population characteristics by HIV risk status

Among the participants, 4,277 (25.8%) were at a high risk of HIV infection (Table 2). Compared with the HIV infection low-risk populations, the HIV infection vulnerable populations were more likely to be male, younger, non-Hispanic White people. Additionally, they were also more likely to have lower income and less married or living together, with higher alcohol drinking levels, cigarette smoking, and a higher occurrence of depression (*p* < 0.05). However, relative to the HIV infection vulnerable populations, the higher BMI, hypertension, and DM were more prevalent among HIV infection low-risk populations (*p* < 0.05). Moreover, no statistically significant difference was observed in the proportions of people who were diagnosed with CVD or CKD, and education level between these two groups.

**Table 2:** Characteristics of participants by HIV risk status in the National Health and Nutrition Examination Survey (NHANES), 1999–2018.

Characteristics	HIV high risk	HIV low risk	*p* value	*N*
	*N = 4,277*	*N = 12,307*		
Age, years, mean (SD)	35.1 (12.1)	39.0 (11.7)	<0.001	16,584
Gender, *N* (%)			<0.001	16,584
Female	2,301 (53.8%)	7,405 (60.2%)		
Male	1,976 (46.2%)	4,902 (39.8%)		
Family income-to-poverty ratio, *N* (%)			<0.001	15,447
<130%	1,518 (38.0%)	3,606 (31.5%)		
>350%	1,072 (26.9%)	3,791 (33.1%)		
130 to <350%	1,402 (35.1%)	4,058 (35.4%)		
Race and ethnicity, *N* (%)			<0.001	16,584
Non-Hispanic white	1,673 (39.1%)	4,999 (40.6%)		
Non-Hispanic black	1,291 (30.2%)	2,339 (19.0%)		
Mexican American	556 (13.0%)	2,250 (18.3%)		
Other Hispanic	387 (9.05%)	1,286 (10.4%)		
Other	370 (8.65%)	1,433 (11.6%)		
Marital status, *N* (%)			<0.001	15,752
Married/living together	1,445 (36.8%)	8,173 (69.1%)		
Never married	1,622 (41.3%)	2,070 (17.5%)		
Widowed/divorced/separated	862 (21.9%)	1,580 (13.4%)		
Education, *N* (%)			0.319	16,584
High school or less	1,886 (44.1%)	5,317 (43.2%)		
More than high school	2,391 (55.9%)	6,990 (56.8%)		
BMI, kg/m^2^, mean (SD)	28.6 (7.34)	29.3 (7.31)	<0.001	16,475
Cerebrovascular disease status, *N* (%)			0.056	15,760
No	3,744 (95.2%)	11,352 (96.0%)		
Yes	187 (4.76%)	477 (4.03%)		
Hypertension status, *N* (%)			0.012	16,584
No	3,205 (74.9%)	8,977 (72.9%)		
Yes	1,072 (25.1%)	3,330 (27.1%)		
Diabetes mellitus status, *N* (%)			<0.001	16,034
Diabetes mellitus	328 (7.87%)	1,343 (11.3%)		
Impaired fasting glucose	118 (2.83%)	399 (3.36%)		
Impaired glucose tolerance	105 (2.52%)	457 (3.85%)		
No	3,618 (86.8%)	9,666 (81.5%)		
Chronic kidney diseases status, *N* (%)			0.466	15,820
No	3,698 (91.1%)	10,668 (90.7%)		
Yes	361 (8.89%)	1,093 (9.29%)		
Depression status, *N* (%)			<0.001	16,584
No	3,720 (87.0%)	11,255 (91.5%)		
Yes	557 (13.0%)	1,052 (8.55%)		
Smoking status, *N* (%)			<0.001	16,137
No	2,634 (64.8%)	9,587 (79.4%)		
Yes	1,433 (35.2%)	2,483 (20.6%)		
Alcohol drinking status, *N* (%)			<0.001	16,362
Former drinker	408 (9.78%)	1,719 (14.1%)		
Heavy drinker	1,636 (39.2%)	2,824 (23.2%)		
Moderate drinker	819 (19.6%)	2,140 (17.6%)		
Mild drinker	1,034 (24.8%)	3,735 (30.6%)		
Never drinker	276 (6.61%)	1,771 (14.5%)		

Data were presented as unweighted frequencies and proportions.

Similar results were also shown in Supplementary Tables 1–5. Participants were classified into five groups (Mexican American, non-Hispanic White, non-Hispanic Black, other Hispanic, or other people) by HIV risk status and gender to assess the correlation between HIV infection vulnerable populations and depression. Relative to the HIV infection low-risk populations, the HIV infection vulnerable populations were more likely to be younger, less married or living together, with higher alcohol drinking levels and smoking status (*p* < 0.05). Additionally, in non-Hispanic White, non-Hispanic Black and other Hispanic people, the HIV infection vulnerable females have higher occurrence of depression than HIV infection low-risk females (*p* < 0.05).

### The association between HIV infection vulnerable populations and depression

To further evaluate the association between HIV infection vulnerable populations and depression. The ORs for depression by HIV risk status were presented in Table 3. According to logistic regression models, the HIV infection vulnerable groups were significantly more likely to suffer depression than control groups with low HIV risk in the pooled sample (*p* < 0.01). After adjusting for age and gender, comparing with those in HIV infection vulnerable participants, those in HIV infection low-risk participants had lower unweighted and weighted odds of depression [OR = 0.55 (0.50–0.62)] and [OR = 0.52 (0.44–0.62)], respectively (*p* < 0.01). In addition, after additionally adjusting for poverty income ratio level, race/ethnicity, BMI, smoking, marital status, and drinking status, relative to the HIV infection vulnerable participants, those in HIV infection low-risk participants also had lower unweighted and weighted odds of depression [OR = 0.69 (0.61–0.79)] and [OR = 0.65 (0.54–0.79), *p* < 0.01]. Moreover, the odds of depression were significantly increased in HIV infection vulnerable group (*p* < 0.01) after further adjusting for the history of CVD, history of hypertension, and history of DM and CKD.

**Table 3:** Association between depression and HIV risk status in the National Health and Nutrition Examination Survey (NHANES), 1999–2018.

Characteristics	Unweighted			Weighted		
	OR (95% CI)		*p* value	OR (95% CI)		*p* value
Model 1^a^						
HIV high risk	1[Reference]			1[Reference]		
HIV low risk	0.55 (0.50–0.62)		<0.001	0.52 (0.44–0.62)		<0.001
Model 2^a^^,^^b^						
HIV high risk	1[Reference]			1[Reference]		
HIV low risk	0.69 (0.61–0.79)		<0.001	0.65 (0.54–0.79)		<0.001
Model 3^a^^,^^b^^,^^c^						
HIV high risk	1[Reference]			1[Reference]		
HIV low risk	0.69 (0.60–0.79)		<0.001	0.64 (0.52–0.78)		<0.001

OR, odds ratio; CI, confidence interval.

a Multivariable-adjusted models were adjusted for age and gender.

b Multivariable-adjusted models were additionally adjusted for poverty income ratio level, race/ethnicity, body mass index, smoking, marital status, and drinking status.

c Multivariable-adjusted models were further adjusted for cerebrovascular disease, hypertension, diabetes mellitus, and chronic kidney disease.

## Discussion

This cross-sectional survey researched the association between depression and HIV infection vulnerable populations in United States adults from 1999 until 2018. More than 25% of the participants were reported to be at high risk of HIV infection, and a tenth had depression. Individuals with a high risk of HIV were male, non-Hispanic White people with lower income, less married or living together, a higher occurrence of depression, and higher levels of cigarette smoking and alcohol drinking. In addition, individuals with depression had a higher occurrence of CVD, hypertension, DM and CKD, and occupied a higher proportion of the HIV infection vulnerable populations. Finally, a multivariable logistic regression model demonstrated that depression might be associated with HIV infection vulnerable populations among the United States adults.

Mental health disorders, especially depression, have been extensively documented in PLWH. A systematic review of 41 pieces of literatures selected from PubMed (June 2018) reported that depression is the most common mental health disease in PLWH, next to drug abuse (29). Various factors contribute to the high incidence of depression, including sadness and sorrow, the worry of living with chronic diseases, difficulty obtaining needed social resources (for example, insurance, finances, and transportation), and internalized stigma and discrimination (1, 12, 29, 30). In addition, PLWH have a high incidence of psychosocial risk for depression caused by early childhood trauma, sexual abuse, unsupportive communities, familial attitudes, exposure to violence, poverty, limited healthcare access, less education, and unemployment (31). Behaviors, which increase the risk of HIV infection and spread include drug and alcohol abuse and adverse mental health conditions (32). HIV-linked clinical causes, such as poor antiretroviral adherence and risky sexual behaviors, are associated with depression (7).

Men who had sex with men continue to be at a very high risk of HIV transmission. In 2018, about 1 million people in the United States were diagnosed HIV infections, with MSM accounting for around 69% of all PLWH (33). Common features of this populations include various forms of stigma (33), unprotected anal intercourse, young age, depression, other mental disorders, substance abuse, and condomless sex factors, those factors have increased the risk of HIV transmission in United States (1, 27, 34). In the cross-sectional analyses on 11,024 participants in the United States found that males were at a higher risk of HIV infection than females, as well as those with a higher educational status compared to those who had a lower educational status. Furthermore, those with a higher income status were at a lower risk of HIV infection compared to those who had a lower income status (2). Similar trends were observed in our study. This analysis confirmed that HIV infection vulnerable populations were male, younger, had lower income, higher levels of cigarette smoking and alcohol drinking, and a higher prevalence of depression than HIV infection low-risk populations. Therefore, this study’s findings suggested that interventions to address adverse mental health conditions and promote healthy lifestyles might prevent subsequent HIV high-risk behaviors.

In this study, we also observed that severe depression and moderately severe depression occupied a higher proportion of the HIV infection vulnerable populations. This aligns with previous research conducted on sexually active male sex workers in the United States, which reported that 74% of male sex workers experienced depressive symptoms and were at a higher risk of engaging in HIV sexual risk behaviors with male clients (19). Similar trends were also found in a cross-sectional epidemiologic study on 300 MSM from the Tanzania observed that depression rates were significantly higher among HIV seropositive MSM compared to HIV seronegative MSM (PR = 1.84, CI = 1.36–2.48), which also found that depression was associated with abuse, HIV, and the risk of HIV transmission (16). Additionally, a previous analysis in the United States, which observed that Hispanic and non-Hispanic Black people had a higher risk of HIV infection than non-Hispanic White people (2). However, our study found that the HIV infection vulnerable populations were more likely to be non-Hispanic White people. These findings might be connected with discrepancies in healthcare access, a higher incidence of other STDs, and partnerships are associated with HIV risk behaviors (35, 36). Another study reported that depression and other adverse mental conditions are more common among White people because drug abuse is more prevalent among White people than Black people (37).

The exact mechanism of causal association between depressive symptoms and HIV infection vulnerable populations remains unclear. However, there are some probable hypotheses. Firstly, depression has been reported to be related to unhealthy behaviors, including smoking, drinking alcohol, condomless sex, substance use, poor nutrition, and lack of physical activity, those factors may contribute to a higher risk of HIV infection. Secondly, the common risk factors of the two populations were lower income, less education, less married, unemployment, and difficulty in getting the needed social resources (for example, insurance, finances, and transportation) (36, 38–41). Thirdly, people with depression may lack the knowledge, social networks, interpersonal skills, impulse control, and inhibitions that may reduce HIV high-risk behaviors (20). Regardless, people with depression should be identified as HIV infection vulnerable populations in prevention arrangement and targeted for HIV prevention efforts (2). Further studies are needed to better understand the complicated associations between depression and HIV infection vulnerable populations.

### Strengths and limitations of this research

Major strengths of this research are based on a large sample size of a well-founded nationwide troop in the United States. This large sample size allowed us to perform joint and stratified analyses with adequate statistical power. Additionally, our study built overall HIV risk status and depressive symptoms measure scores to comprehensively assess the complex relationships between depression and the HIV infection vulnerable populations. Moreover, a series of covariate analyses were constructed in this study to present our finding that depression might be associated with HIV infection vulnerable populations.

This study also had some limitations. Firstly, data on depressive symptoms and HIV risk behaviors were mostly self-reported. Secondly, lifestyle varies during adulthood, but this study could not obtain long-term HIV risk status routes. Thirdly, selection bias might have occurred because of those excluded from analyses due to lost data for HIV risk behaviors and PHQ-9. Lastly, the causal relationship between depression and HIV infection vulnerable populations could not be determined owing to the cross-sectional nature of the observational research.

## Conclusion

Based on a large nationwide United States cohort, depression might be associated with HIV infection vulnerable populations in the United States adults. More research is needed to evaluate the association between HIV infection vulnerable populations and depression, as well as their causal associations. In addition, the prevention efforts focusing on HIV disclosure and HIV infection vulnerable populations in the United States must address common co-prevalent depression to reduce the rate of new HIV infections.

## Data availability statement

The original contributions presented in the study are included in the article/Supplementary material, further inquiries can be directed to the corresponding authors.

## Ethics statement

Ethical approval for this research was considered free because it adopted openly obtainable second-hand data.

## Author contributions

ZX and BL: conceptualization, methodology, data analysis, formal management, and visualization. YX and YH: drafting of the manuscript. JP and RT: writing, review, and editing. All authors contributed to the article and approved the submitted version.

## Funding

The authors received funding from the Natural Science Foundation of Changsha Project (kq2202487) and Scientific Research Project of Hunan Aerospace Hospital (2022yj19).

## Conflict of interest

The authors declare that the research was conducted in the absence of any commercial or financial relationships that could be construed as a potential conflict of interest.

## Publisher’s note

All claims expressed in this article are solely those of the authors and do not necessarily represent those of their affiliated organizations, or those of the publisher, the editors and the reviewers. Any product that may be evaluated in this article, or claim that may be made by its manufacturer, is not guaranteed or endorsed by the publisher.
