# Supplementary material for: Association between depression and HIV infection vulnerable populations in United States adults: a cross-sectional analysis of NHANES from 1999 to 2018
Source: Front Public Health. 2023 Jun 1;11:1146318. doi: 10.3389/fpubh.2023.1146318 (PMC10267355; doi:10.3389/fpubh.2023.1146318)
Supplement: Supplementary file 1 [file Table_1.docx]

**Supplementary Table 1. Characteristics of Non-Hispanic White people by HIV risk** **status and gender in the National Health and Nutrition Examination Survey (NHANES), 1999–2018.**

| **Characteristics** | **Female** | | | **Male** | | |
| --- | --- | --- | --- | --- | --- | --- |
|  | **HIV high risk** | **HIV low risk** | ***p*-value** | **HIV high risk** | **HIV low risk** | ***p*-value** |
|  | ***N=976*** | ***N=2962*** |  | ***N=697*** | ***N=2037*** |  |
| **Age, years, mean (SD)** | 36.2 (11.8) | 39.8 (11.4) | <0.001 | 36.7 (12.4) | 39.6 (11.6) | <0.001 |
| **Family income-to-poverty ratio, N (%)** |  |  | <0.001 |  |  | 0.001 |
| <130% | 328 (34.8%) | 740 (25.9%) |  | 237 (35.5%) | 553 (28.2%) |  |
| >350% | 338 (35.9%) | 1217 (42.6%) |  | 228 (34.2%) | 800 (40.9%) |  |
| 130% to <350% | 276 (29.3%) | 903 (31.6%) |  | 202 (30.3%) | 605 (30.9%) |  |
| **Marital status, N (%)** |  |  | <0.001 |  |  | <0.001 |
| Married/living together | 421 (45.3%) | 2106 (72.2%) |  | 224 (34.6%) | 1442 (73.3%) |  |
| Never married | 256 (27.6%) | 360 (12.3%) |  | 266 (41.0%) | 333 (16.9%) |  |
| Widowed/divorced/separated | 252 (27.1%) | 449 (15.4%) |  | 158 (24.4%) | 192 (9.76%) |  |
| **Education, N (%)** |  |  | 0.747 |  |  | 0.007 |
| High school or less | 324 (33.2%) | 1002 (33.8%) |  | 307 (44.0%) | 778 (38.2%) |  |
| More than high school | 652 (66.8%) | 1960 (66.2%) |  | 390 (56.0%) | 1259 (61.8%) |  |
| **BMI, kg/m^2^, mean (SD)** | 28.2 (7.70) | 28.9 (7.66) | 0.013 | 27.4 (5.68) | 28.9 (6.49) | <0.001 |
| **Cerebrovascular disease status, N (%)** |  |  | 0.883 |  |  | 0.204 |
| No | 889 (95.7%) | 2786 (95.5%) |  | 610 (94.1%) | 1878 (95.5%) |  |
| Yes | 40 (4.31%) | 131 (4.49%) |  | 38 (5.86%) | 89 (4.52%) |  |
| **Hypertension status, N (%)** |  |  | 0.615 |  |  | 0.035 |
| No | 754 (77.3%) | 2263 (76.4%) |  | 508 (72.9%) | 1396 (68.5%) |  |
| Yes | 222 (22.7%) | 699 (23.6%) |  | 189 (27.1%) | 641 (31.5%) |  |
| **Diabetes mellitus status, N (%)** |  |  | 0.001 |  |  | 0.097 |
| Diabetes mellitus | 53 (5.66%) | 247 (8.88%) |  | 52 (7.46%) | 187 (9.18%) |  |
| Impaired fasting glucose | 13 (1.39%) | 70 (2.52%) |  | 33 (4.73%) | 109 (5.35%) |  |
| Impaired glucose tolerance | 27 (2.88%) | 103 (3.70%) |  | 12 (1.72%) | 62 (3.04%) |  |
| No | 843 (90.1%) | 2362 (84.9%) |  | 600 (86.1%) | 1679 (82.4%) |  |
| **Chronic kidney diseases status, N (%)** |  |  | 0.206 |  |  | 0.585 |
| No | 858 (91.9%) | 2584 (90.4%) |  | 621 (93.1%) | 1846 (93.8%) |  |
| Yes | 76 (8.14%) | 274 (9.59%) |  | 46 (6.90%) | 122 (6.20%) |  |
| **Depression status, N (%)** |  |  | <0.001 |  |  | 0.095 |
| No | 793 (81.2%) | 2660 (89.8%) |  | 632 (90.7%) | 1889 (92.7%) |  |
| Yes | 183 (18.8%) | 302 (10.2%) |  | 65 (9.33%) | 148 (7.27%) |  |
| **Smoking status, N (%)** |  |  | <0.001 |  |  | <0.001 |
| No | 563 (59.2%) | 2172 (74.0%) |  | 359 (54.0%) | 1459 (73.2%) |  |
| Yes | 388 (40.8%) | 763 (26.0%) |  | 306 (46.0%) | 534 (26.8%) |  |
| **Alcohol drinking status, N (%)** |  |  | <0.001 |  |  | <0.001 |
| Former drinker | 105 (10.9%) | 426 (14.5%) |  | 77 (11.4%) | 280 (13.9%) |  |
| Heavy drinker | 381 (39.7%) | 637 (21.6%) |  | 323 (47.7%) | 586 (29.1%) |  |
| Moderate drinker | 248 (25.8%) | 725 (24.6%) |  | 79 (11.7%) | 256 (12.7%) |  |
| Mild drinker | 192 (20.0%) | 831 (28.2%) |  | 181 (26.7%) | 787 (39.0%) |  |
| Never drinker | 34 (3.54%) | 326 (11.1%) |  | 17 (2.51%) | 108 (5.35%) |  |

Data were presented as unweighted frequencies and proportions.

**Supplementary Table 2. Characteristics of Non-Hispanic Black people by HIV risk** **status and gender in the National Health and Nutrition Examination Survey (NHANES), 1999–2018.**

| **Characteristics** | **Female** | | | **Male** | | | |
| --- | --- | --- | --- | --- | --- | --- | --- |
|  | **HIV high risk** | **HIV low risk** | ***p*-value** | | **HIV high risk** | **HIV low risk** | ***p*-value** |
|  | ***N=679*** | ***N=1490*** |  | | ***N=612*** | ***N=849*** |  |
| **Age, years, mean (SD)** | 34.0 (11.6) | 40.1 (12.1) | <0.001 | | 35.2 (12.9) | 39.7 (12.2) | <0.001 |
| **Family income-to-poverty ratio, N (%)** |  |  | <0.001 | |  |  | <0.001 |
| <130% | 270 (42.7%) | 468 (33.8%) |  | | 228 (41.0%) | 230 (29.9%) |  |
| >350% | 108 (17.1%) | 379 (27.3%) |  | | 123 (22.1%) | 224 (29.1%) |  |
| 130% to <350% | 255 (40.3%) | 539 (38.9%) |  | | 205 (36.9%) | 316 (41.0%) |  |
| **Marital status, N (%)** |  |  | <0.001 | |  |  | <0.001 |
| Married/living together | 156 (25.0%) | 650 (45.9%) |  | | 149 (27.5%) | 521 (64.7%) |  |
| Never married | 345 (55.2%) | 459 (32.4%) |  | | 286 (52.9%) | 201 (25.0%) |  |
| Widowed/divorced/separated | 124 (19.8%) | 308 (21.7%) |  | | 106 (19.6%) | 83 (10.3%) |  |
| **Education, N (%)** |  |  | 0.109 | |  |  | 0.024 |
| High school or less | 280 (41.2%) | 559 (37.5%) |  | | 333 (54.4%) | 410 (48.3%) |  |
| More than high school | 399 (58.8%) | 931 (62.5%) |  | | 279 (45.6%) | 439 (51.7%) |  |
| **BMI, kg/m^2^, mean (SD)** | 31.4 (9.01) | 32.3 (8.82) | 0.027 | | 28.1 (6.83) | 29.8 (7.53) | <0.001 |
| **Cerebrovascular disease status, N (%)** |  |  | 0.301 | |  |  | 1.000 |
| No | 593 (94.9%) | 1327 (93.6%) |  | | 512 (94.6%) | 764 (94.8%) |  |
| Yes | 32 (5.12%) | 91 (6.42%) |  | | 29 (5.36%) | 42 (5.21%) |  |
| **Hypertension status, N (%)** |  |  | <0.001 | |  |  | 0.009 |
| No | 483 (71.1%) | 902 (60.5%) |  | | 411 (67.2%) | 512 (60.3%) |  |
| Yes | 196 (28.9%) | 588 (39.5%) |  | | 201 (32.8%) | 337 (39.7%) |  |
| **Diabetes mellitus status, N (%)** |  |  | 0.001 | |  |  | 0.014 |
| Diabetes mellitus | 61 (9.49%) | 209 (14.7%) |  | | 56 (9.15%) | 121 (14.3%) |  |
| Impaired fasting glucose | 13 (2.02%) | 22 (1.54%) |  | | 17 (2.78%) | 31 (3.65%) |  |
| Impaired glucose tolerance | 8 (1.24%) | 40 (2.81%) |  | | 19 (3.10%) | 19 (2.24%) |  |
| No | 561 (87.2%) | 1154 (81.0%) |  | | 520 (85.0%) | 678 (79.9%) |  |
| **Chronic kidney diseases status, N (%)** |  |  | 0.642 | |  |  | 0.467 |
| No | 563 (88.0%) | 1190 (87.1%) |  | | 513 (90.5%) | 695 (89.1%) |  |
| Yes | 77 (12.0%) | 176 (12.9%) |  | | 54 (9.52%) | 85 (10.9%) |  |
| **Depression status, N (%)** |  |  | 0.001 | |  |  | 0.647 |
| No | 571 (84.1%) | 1330 (89.3%) |  | | 567 (92.6%) | 793 (93.4%) |  |
| Yes | 108 (15.9%) | 160 (10.7%) |  | | 45 (7.35%) | 56 (6.60%) |  |
| **Smoking status, N (%)** |  |  | <0.001 | |  |  | <0.001 |
| No | 478 (73.9%) | 1178 (81.1%) |  | | 346 (61.0%) | 588 (71.0%) |  |
| Yes | 169 (26.1%) | 275 (18.9%) |  | | 221 (39.0%) | 240 (29.0%) |  |
| **Alcohol drinking status, N (%)** |  |  | <0.001 | |  |  | <0.001 |
| Former drinker | 68 (10.1%) | 223 (15.1%) |  | | 49 (8.28%) | 104 (12.4%) |  |
| Heavy drinker | 169 (25.2%) | 217 (14.7%) |  | | 183 (30.9%) | 179 (21.3%) |  |
| Moderate drinker | 171 (25.5%) | 312 (21.2%) |  | | 84 (14.2%) | 114 (13.6%) |  |
| Mild drinker | 177 (26.4%) | 414 (28.1%) |  | | 230 (38.9%) | 342 (40.8%) |  |
| Never drinker | 85 (12.7%) | 309 (20.9%) |  | | 46 (7.77%) | 100 (11.9%) |  |

Data were presented as unweighted frequencies and proportions.

**Supplementary Table 3. Characteristics of Mexican American by HIV risk** **status and gender in the National Health and Nutrition Examination Survey (NHANES), 1999–2018.**

| **Characteristics** | **Female** | | | **Male** | | |
| --- | --- | --- | --- | --- | --- | --- |
|  | **HIV high risk** | **HIV low risk** | ***p*-value** | **HIV high risk** | **HIV low risk** | ***p*-value** |
|  | ***N=275*** | ***N=1399*** |  | ***N=281*** | ***N=851*** |  |
| **Age, years, mean (SD)** | 34.2 (11.7) | 37.0 (11.5) | <0.001 | 34.7 (12.7) | 37.4 (11.9) | 0.002 |
| **Family income-to-poverty ratio, N (%)** |  |  | 0.871 |  |  | 0.078 |
| <130% | 119 (47.2%) | 574 (46.0%) |  | 91 (36.1%) | 334 (43.7%) |  |
| >350% | 42 (16.7%) | 202 (16.2%) |  | 43 (17.1%) | 102 (13.4%) |  |
| 130% to <350% | 91 (36.1%) | 473 (37.9%) |  | 118 (46.8%) | 328 (42.9%) |  |
| **Marital status, N (%)** |  |  | <0.001 |  |  | <0.001 |
| Married/living together | 114 (46.3%) | 970 (73.0%) |  | 111 (45.5%) | 623 (79.1%) |  |
| Never married | 72 (29.3%) | 168 (12.7%) |  | 89 (36.5%) | 109 (13.8%) |  |
| Widowed/divorced/separated | 60 (24.4%) | 190 (14.3%) |  | 44 (18.0%) | 56 (7.11%) |  |
| **Education, N (%)** |  |  | 0.029 |  |  | 0.076 |
| High school or less | 164 (59.6%) | 933 (66.7%) |  | 191 (68.0%) | 627 (73.7%) |  |
| More than high school | 111 (40.4%) | 466 (33.3%) |  | 90 (32.0%) | 224 (26.3%) |  |
| **BMI, kg/m^2^, mean (SD)** | 29.9 (7.27) | 30.3 (6.98) | 0.447 | 28.5 (5.79) | 29.7 (5.72) | 0.005 |
| **Cerebrovascular disease status, N (%)** |  |  | 0.031 |  |  | 0.099 |
| No | 235 (95.5%) | 1303 (98.0%) |  | 230 (94.3%) | 764 (96.8%) |  |
| Yes | 11 (4.47%) | 26 (1.96%) |  | 14 (5.74%) | 25 (3.17%) |  |
| **Hypertension status, N (%)** |  |  | 0.684 |  |  | 0.729 |
| No | 226 (82.2%) | 1132 (80.9%) |  | 223 (79.4%) | 665 (78.1%) |  |
| Yes | 49 (17.8%) | 267 (19.1%) |  | 58 (20.6%) | 186 (21.9%) |  |
| **Diabetes mellitus status, N (%)** |  |  | 0.061 |  |  | 0.095 |
| Diabetes mellitus | 22 (8.56%) | 172 (13.4%) |  | 30 (10.7%) | 118 (13.9%) |  |
| Impaired fasting glucose | 6 (2.33%) | 25 (1.95%) |  | 11 (3.91%) | 53 (6.23%) |  |
| Impaired glucose tolerance | 11 (4.28%) | 85 (6.64%) |  | 5 (1.78%) | 27 (3.17%) |  |
| No | 218 (84.8%) | 998 (78.0%) |  | 235 (83.6%) | 653 (76.7%) |  |
| **Chronic kidney diseases status, N (%)** |  |  | 0.628 |  |  | 0.45 |
| No | 234 (90.0%) | 1206 (88.7%) |  | 258 (94.2%) | 760 (92.6%) |  |
| Yes | 26 (10.0%) | 153 (11.3%) |  | 16 (5.84%) | 61 (7.43%) |  |
| **Depression status, N (%)** |  |  | 0.052 |  |  | 0.002 |
| No | 239 (86.9%) | 1272 (90.9%) |  | 257 (91.5%) | 820 (96.4%) |  |
| Yes | 36 (13.1%) | 127 (9.08%) |  | 24 (8.54%) | 31 (3.64%) |  |
| **Smoking status, N (%)** |  |  | 0.005 |  |  | <0.001 |
| No | 219 (84.9%) | 1244 (90.8%) |  | 179 (68.8%) | 661 (80.9%) |  |
| Yes | 39 (15.1%) | 126 (9.20%) |  | 81 (31.2%) | 156 (19.1%) |  |
| **Alcohol drinking status, N (%)** |  |  | <0.001 |  |  | <0.001 |
| Former drinker | 32 (12.0%) | 214 (15.5%) |  | 23 (8.61%) | 118 (14.2%) |  |
| Heavy drinker | 108 (40.4%) | 306 (22.1%) |  | 160 (59.9%) | 361 (43.5%) |  |
| Moderate drinker | 57 (21.3%) | 250 (18.1%) |  | 42 (15.7%) | 99 (11.9%) |  |
| Mild drinker | 36 (13.5%) | 264 (19.1%) |  | 36 (13.5%) | 201 (24.2%) |  |
| Never drinker | 34 (12.7%) | 348 (25.2%) |  | 6 (2.25%) | 50 (6.03%) |  |

Data were presented as unweighted frequencies and proportions.

**Supplementary Table 4. Characteristics of Other Hispanic people by HIV risk** **status and gender in the National Health and Nutrition Examination Survey (NHANES), 1999–2018.**

| **Characteristics** | **Female** | | | **Male** | | |
| --- | --- | --- | --- | --- | --- | --- |
|  | **HIV high risk** | **HIV low risk** | ***p*-overall** | **HIV high risk** | **HIV low risk** | ***p*-overall** |
|  | ***N=193*** | ***N=805*** |  | ***N=194*** | ***N=481*** |  |
| **Age, years, mean (SD)** | 33.9 (11.3) | 38.9 (12.2) | <0.001 | 35.2 (12.5) | 38.8 (11.1) | <0.001 |
| **Family income-to-poverty ratio, N (%)** |  |  | 0.119 |  |  | 0.135 |
| <130% | 75 (42.9%) | 264 (36.7%) |  | 70 (41.7%) | 149 (35.1%) |  |
| >350% | 29 (16.6%) | 167 (23.2%) |  | 42 (25.0%) | 97 (22.8%) |  |
| 130% to <350% | 71 (40.6%) | 288 (40.1%) |  | 56 (33.3%) | 179 (42.1%) |  |
| **Marital status, N (%)** |  |  | <0.001 |  |  | <0.001 |
| Married/living together | 83 (45.9%) | 494 (65.0%) |  | 73 (40.3%) | 335 (72.7%) |  |
| Never married | 62 (34.3%) | 127 (16.7%) |  | 76 (42.0%) | 77 (16.7%) |  |
| Widowed/divorced/separated | 36 (19.9%) | 139 (18.3%) |  | 32 (17.7%) | 49 (10.6%) |  |
| **Education, N (%)** |  |  | 0.282 |  |  | 0.099 |
| High school or less | 92 (47.7%) | 421 (52.3%) |  | 93 (47.9%) | 266 (55.3%) |  |
| More than high school | 101 (52.3%) | 384 (47.7%) |  | 101 (52.1%) | 215 (44.7%) |  |
| **BMI, kg/m^2^, mean (SD)** | 29.2 (7.70) | 28.8 (6.87) | 0.54 | 27.9 (5.57) | 29.1 (5.64) | 0.009 |
| **Cerebrovascular disease status, N (%)** |  |  | 1 |  |  | 0.895 |
| No | 174 (96.1%) | 732 (96.3%) |  | 175 (96.2%) | 446 (96.7%) |  |
| Yes | 7 (3.87%) | 28 (3.68%) |  | 7 (3.85%) | 15 (3.25%) |  |
| **Hypertension status, N (%)** |  |  | 0.107 |  |  | 0.855 |
| No | 160 (82.9%) | 622 (77.3%) |  | 150 (77.3%) | 367 (76.3%) |  |
| Yes | 33 (17.1%) | 183 (22.7%) |  | 44 (22.7%) | 114 (23.7%) |  |
| **Diabetes mellitus status, N (%)** |  |  | 0.479 |  |  | 0.006 |
| Diabetes mellitus | 19 (10.1%) | 84 (10.9%) |  | 10 (5.15%) | 64 (13.3%) |  |
| Impaired fasting glucose | 7 (3.72%) | 20 (2.60%) |  | 8 (4.12%) | 27 (5.61%) |  |
| Impaired glucose tolerance | 4 (2.13%) | 32 (4.16%) |  | 10 (5.15%) | 13 (2.70%) |  |
| No | 158 (84.0%) | 634 (82.3%) |  | 166 (85.6%) | 377 (78.4%) |  |
| **Chronic kidney diseases status, N (%)** |  |  | 0.673 |  |  | 0.468 |
| No | 159 (89.3%) | 712 (90.7%) |  | 172 (91.5%) | 430 (93.5%) |  |
| Yes | 19 (10.7%) | 73 (9.30%) |  | 16 (8.51%) | 30 (6.52%) |  |
| **Depression status, N (%)** |  |  | 0.002 |  |  | 0.732 |
| No | 149 (77.2%) | 696 (86.5%) |  | 176 (90.7%) | 442 (91.9%) |  |
| Yes | 44 (22.8%) | 109 (13.5%) |  | 18 (9.28%) | 39 (8.11%) |  |
| **Smoking status, N (%)** |  |  | <0.001 |  |  | 0.008 |
| No | 133 (72.3%) | 690 (87.7%) |  | 130 (69.9%) | 378 (79.9%) |  |
| Yes | 51 (27.7%) | 97 (12.3%) |  | 56 (30.1%) | 95 (20.1%) |  |
| **Alcohol drinking status, N (%)** |  |  | <0.001 |  |  | <0.001 |
| Former drinker | 22 (11.6%) | 117 (14.6%) |  | 12 (6.28%) | 72 (15.1%) |  |
| Heavy drinker | 77 (40.7%) | 167 (20.9%) |  | 99 (51.8%) | 169 (35.4%) |  |
| Moderate drinker | 36 (19.0%) | 160 (20.0%) |  | 31 (16.2%) | 60 (12.6%) |  |
| Mild drinker | 31 (16.4%) | 177 (22.1%) |  | 42 (22.0%) | 135 (28.3%) |  |
| Never drinker | 23 (12.2%) | 179 (22.4%) |  | 7 (3.66%) | 41 (8.60%) |  |

Data were presented as unweighted frequencies and proportions.

**Supplementary Table 5. Characteristics of Other people by HIV risk** **status and gender in the National Health and Nutrition Examination Survey (NHANES), 1999–2018.**

| **Characteristics** | **Female** | | | **Male** | | |
| --- | --- | --- | --- | --- | --- | --- |
|  | **HIV high risk** | **HIV low risk** | ***p*-value** | **HIV high risk** | **HIV low risk** | ***p*-value** |
|  | ***N=178*** | ***N=749*** |  | ***N=192*** | ***N=684*** |  |
| **Age, years, mean (SD)** | 32.9 (11.1) | 38.0 (11.2) | <0.001 | 31.8 (10.7) | 38.6 (11.3) | <0.001 |
| **Family income-to-poverty ratio, N (%)** |  |  | 0.004 |  |  | 0.073 |
| <130% | 56 (32.6%) | 158 (22.6%) |  | 44 (25.1%) | 136 (21.7%) |  |
| >350% | 56 (32.6%) | 317 (45.4%) |  | 63 (36.0%) | 286 (45.7%) |  |
| 130% to <350% | 60 (34.9%) | 223 (31.9%) |  | 68 (38.9%) | 204 (32.6%) |  |
| **Marital status, N (%)** |  |  | <0.001 |  |  | <0.001 |
| Married/living together | 59 (36.2%) | 535 (74.1%) |  | 55 (32.2%) | 497 (75.3%) |  |
| Never married | 74 (45.4%) | 114 (15.8%) |  | 96 (56.1%) | 122 (18.5%) |  |
| Widowed/divorced/separated | 30 (18.4%) | 73 (10.1%) |  | 20 (11.7%) | 41 (6.21%) |  |
| **Education, N (%)** |  |  | 0.341 |  |  | 0.094 |
| High school or less | 43 (24.2%) | 154 (20.6%) |  | 59 (30.7%) | 167 (24.4%) |  |
| More than high school | 135 (75.8%) | 595 (79.4%) |  | 133 (69.3%) | 517 (75.6%) |  |
| **BMI, kg/m^2^, mean (SD)** | 26.8 (7.86) | 25.9 (6.70) | 0.154 | 26.0 (5.20) | 26.6 (5.69) | 0.206 |
| **Cerebrovascular disease status, N (%)** |  |  | 1.000 |  |  | 0.251 |
| No | 160 (98.2%) | 705 (97.6%) |  | 166 (96.5%) | 647 (98.0%) |  |
| Yes | 3 (1.84%) | 17 (2.35%) |  | 6 (3.49%) | 13 (1.97%) |  |
| **Hypertension status, N (%)** |  |  | 0.928 |  |  | 0.731 |
| No | 140 (78.7%) | 594 (79.3%) |  | 150 (78.1%) | 524 (76.6%) |  |
| Yes | 38 (21.3%) | 155 (20.7%) |  | 42 (21.9%) | 160 (23.4%) |  |
| **Diabetes mellitus status, N (%)** |  |  | 0.054 |  |  | 0.054 |
| Diabetes mellitus | 14 (8.28%) | 72 (10.2%) |  | 11 (5.73%) | 69 (10.1%) |  |
| Impaired fasting glucose | 6 (3.55%) | 12 (1.70%) |  | 4 (2.08%) | 30 (4.39%) |  |
| Impaired glucose tolerance | 4 (2.37%) | 46 (6.52%) |  | 5 (2.60%) | 30 (4.39%) |  |
| No | 145 (85.8%) | 576 (81.6%) |  | 172 (89.6%) | 555 (81.1%) |  |
| **Chronic kidney diseases status, N (%)** |  |  | 0.635 |  |  | 0.723 |
| No | 150 (89.3%) | 645 (90.8%) |  | 170 (92.9%) | 600 (91.7%) |  |
| Yes | 18 (10.7%) | 65 (9.15%) |  | 13 (7.10%) | 54 (8.26%) |  |
| **Depression status, N (%)** |  |  | 0.073 |  |  | 0.094 |
| No | 157 (88.2%) | 694 (92.7%) |  | 179 (93.2%) | 659 (96.3%) |  |
| Yes | 21 (11.8%) | 55 (7.34%) |  | 13 (6.77%) | 25 (3.65%) |  |
| **Smoking status, N (%)** |  |  | <0.001 |  |  | <0.001 |
| No | 111 (65.3%) | 671 (90.6%) |  | 116 (64.8%) | 546 (81.1%) |  |
| Yes | 59 (34.7%) | 70 (9.45%) |  | 63 (35.2%) | 127 (18.9%) |  |
| **Alcohol drinking status, N (%)** |  |  | <0.001 |  |  | <0.001 |
| Former drinker | 12 (6.86%) | 84 (11.3%) |  | 8 (4.32%) | 81 (11.9%) |  |
| Heavy drinker | 71 (40.6%) | 98 (13.2%) |  | 65 (35.1%) | 104 (15.3%) |  |
| Moderate drinker | 42 (24.0%) | 98 (13.2%) |  | 29 (15.7%) | 66 (9.71%) |  |
| Mild drinker | 36 (20.6%) | 248 (33.3%) |  | 73 (39.5%) | 336 (49.4%) |  |
| Never drinker | 14 (8.00%) | 217 (29.1%) |  | 10 (5.41%) | 93 (13.7%) |  |

Data were presented as unweighted frequencies and proportions.
